# Dr. W. W. Reeves

**Published:** 1891-02

**Authors:** 


DR. W. W. REEVES, the new superintendent of the Texas State Lunatic Asylum at Austin, is a native of Virginia. He is the son of Geo. W. and Caroline Reeves, Americans, and was born June 23, 1847. Received his education at Jefferson High School; studied medicine at Creston, Ash county, N. C., with Dr. J. O. Wilcox; attended lectures at Baltimore and Philadelphia in 1866 and 1869, and again in 1879, graduating that year at Baltimore. He came to Texas in 1870, and located at Cedar Grove. Here he resided till 1874, when he went to Will's Point, VanZandt county. He practiced there continuously, till he was honored by the governor with the appointment of superintendent of the State Lunatic Asylum. He is perhaps the youngest man ever appointed to this responsible position.

Dr. Reeves is a member of the Texas State Medical Association, and at this writing, February, '91, is one of the vice-presidents. He has been for several years a member and secretary of the Board of Medical Examiners for the Seventh Judicial District, and has also been, during the time, surgeon of the Texas and Pacific railroad. He has been twice married. His first wife was Miss Cora A. Horton. His present wife, to whom he was married in 1879, was Miss Margaret Knotzsch. They have three children.

